# Management of Pruritus Ani

**Published:** 1905-01

**Authors:** Henry Rockwell Varney

**Affiliations:** Radiographist and Radiotherapist to Harper Hospital, Attending Physician for Skin Diseases, Harper Hospital Polyclinic


### MANAGEMENT OF PRURITUS ANI.

BY HENRY ROCKWELL VARNEY, M.D.,
Radiographist and Radiotherapist to Harper Hospital, Attending Physician for Skin Diseases, Harper Hospital Polyclinic.

Pruritus ani is a condition that presents many characteristic eccentricities. Its most marked feature is itching, and an itching that is not relieved by scratching, as is usual on other parts of the body. It comes on, as a rule, when the patient is in repose. The incessant itching is out of all proportion to the appearance of the affected part. Time does not palliate the condition; on the contrary, the constant scratching, the usual relief for itching, aggravates it, and makes a cure much more difficult to effect.

The etiological factors that are attributed as the cause of pruritus ani could scarcely be enumerated, yet all of them are no doubt irritations of the peripheral nerves in this region. Almost every disease known has been assigned as the cause of pruritus, and almost every adjective in the English language has been used by the affected patients to describe the persistent irritation. As a rule, uneasiness about the part is first experienced. Soon, however, the itching increases with such rapidity and force that the sufferer is willing to try anything for relief suggested by either physician or laiety, and no condition has had more specifics, both medical and surgical, advanced for its relief. The physician who by chance relieves the sufferer at once will have a most faithful follower in the after-treatment of the condition.

The causes may be external, internal, constitutional, and reflex. In the management of this condition, on the removal of the cause depends the result. Structural changes, caused by scratching, complicate the treatment. Every case of pruritus is a problem in itself.

Pruritus ani, as seen by the dermatologist of today, are usually cases that have been treated both surgically and medically, and the causes have been supposedly removed. While a large percentage of cases are cured in this way, there are those that persist, driving the patient often to the verge of suicide by the torture. The condition is often of years' standing. It matters not to the patient whether pruritus is a disease or a symptom of a pathological condition; any treatment that will relieve the irritation at once, without too much torture to the patient, and give him the needed sleep, and general quieting of the nervous condition, is the one to be selected. With these cases—more especially in the middle-aged and old ("prurigo senilis" of the old writers) the management and study of each individual case should be carried out most carefully, and the prognosis should be guarded.

The first and most important step then is to relieve the itching. Then ascertain if possible, what pathological condition is causing the distress.

In the first aid to the sufferer, local application has been our sheet anchor. This gives temporary relief, as a rule, during which relief we try to determine just what is causing the pruritus, and direct treatment to it, if it is discovered. All local applications act best after the patient has bathed the affected part with hot water, and all irritating underclothing removed. A mild treatment should be suggested first, such as pressure on the part, hot or cold water applications. These furnish this temporary relief. Injections of hot, mild astringent preparations are many times successful. One of the most effective drugs is carbolic acid, in ointment or lotion form, the latter preferred, combined with some cooling, evaporating base. Black wash is an established and well-recommended lotion. In Europe, the most universal preparations are those containing tar. As a last resort, cocaine is sometime used, but it should be given without the patient's knowledge, to guard against an abuse of the drug, and the precaution taken to continue its use only as long as is necessary.

It would be impossible to enumerate the number of remedies one is often obliged to try in many of the obstinate cases, but in my late experience in the treatment of these cases nothing has been more successful than the light treatment (radiotherapy). It is not only quick in its effect, but brings about a change in the cell structure of the part that is more universally permanent than other treatments.

Chronic cases, of thirteen and sixteen years' standing, when so treated, have been completely relieved after three treatments, and after from ten to twenty treatments the patients were discharged.

With histories of the existing conditions for so many years, even the specialist would hesitate before suggesting medications, for everything, both medical and surgical, had been tried until the patients were utterly discouraged.

Recent experience has taught me that the light treatment, properly given, will relieve a large percentage of these chronic cases of pruritus ani. This treatment is by no means new, many operators having for two years past reported equally good results.

After "running the gauntlet" of local applications for the relief of such cases, and after all supposed original causes of the condition have been removed, with no relief to the tormented patient, I feel one can welcome a treatment that, in my experience, has thus far not failed to relieve these cases. All itching about the buttocks is by no means pruritus ani; therefore, one must be most careful in examining the parts to make a correct diagnosis before installing treatment. If symptoms of cutaneous diseases, such as eczema, herpes, dermatitises caused by parasitic affections, and other external irritating agencies, are present, they should not be confused with true pruritus ani.

It is well to try the light treatment before resorting to the actual cautery or caustics, blisters or removal of the skin of the affected part, for the patient suffers no inconvenience from the treatment. Yet in all chronic cases the true cause must be discovered and corrected, if possible, or any local treatment will give only temporary relief. Cases so treated, as with other forms of treatment, may recur, if the original cause of the pruritus is not removed.—*Harper Hospital Bulletin.*

